# Neurofunctional predictive biomarkers of cognitive-behavioral therapy during fear conditioning in patients with obsessive-compulsive disorder

**DOI:** 10.1192/j.eurpsy.2021.376

**Published:** 2021-08-13

**Authors:** M. Cano-Catala, I. Martinez-Zalacain, E. Real, P. Alonso, J.M. Menchon, N. Cardoner Álvarez, M.A. Fullana, C. Soriano-Mas

**Affiliations:** 1 Mental Health Department, Parc Tauli University Hospital, Sabadell, Spain; 2 Department Of Psychiatry, Bellvitge University Hospital, Hospitalet de LLobregat, Spain; 3 Department Of Psychiatry, Hospital Clinic-Institute of Neurosciences, Barcelona, Spain

**Keywords:** Cognitive-behavioral therapy, Fear conditioning, Predictive biomarkers, Obsessive-Compulsive disorder

## Abstract

**Introduction:**

Altered fear learning processes could be mechanistically linked to the development and/or maintenance of obsessive-compulsive disorder (OCD). From a clinical perspective, the first-line psychological treatment for OCD is cognitive-behavioral therapy (CBT), which is based on the principles of fear learning. However, no previous functional magnetic resonance imaging (fMRI) studies have evaluated the predictive capacity of regional brain activations during fear learning on CBT response in patients with OCD.

**Objectives:**

We aimed at exploring whether brain activation during fear learning in patients with OCD are associated with CBT outcome.

**Methods:**

We assessed 18 patients with OCD and 18 healthy participants during a 2-day experimental protocol where brain activation and skin conductance responses (SCR) where assessed during fear conditioning, extinction learning, and extinction recall within the fMRI scanner. Following the protocol, patients with OCD received CBT.

**Results:**

We found non-significant between-group differences in SCR during fear learning. Patients with OCD showed significantly diminished activation of the dorsal anterior cingulate cortex and the right insula during fear conditioning. Importantly, our analyses revealed a significant negative association between clinical improvement after CBT and activity at the right insula during fear conditioning (x = 39, y = 12, z = -11; t = 5.64; p<0.001; k = 928). This finding is displayed in Figure 1 below.

**Figure:**
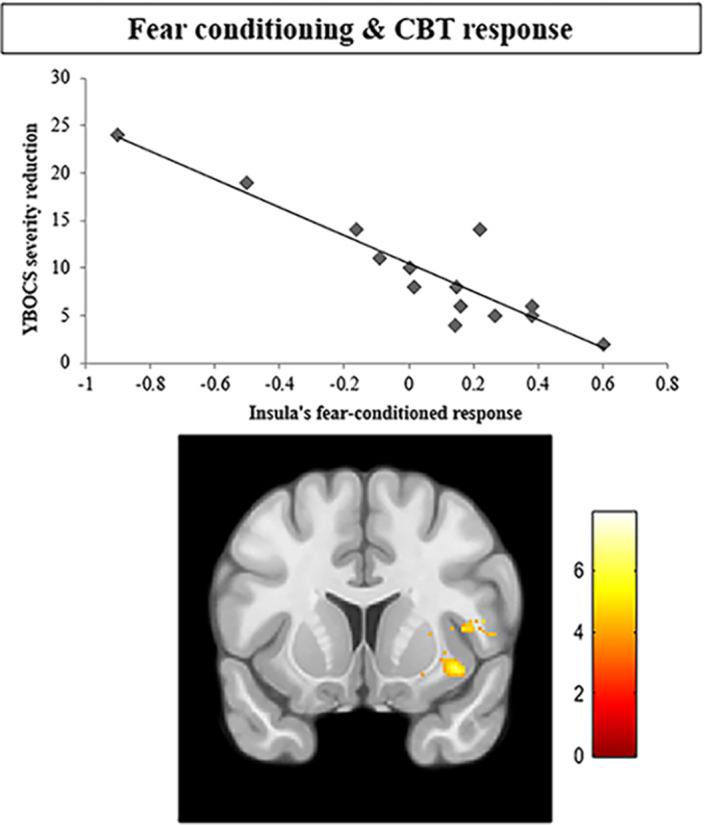

**Conclusions:**

Patients with OCD may require less fear-conditioned brain responses to achieve the same level of psychophysiological fear conditioning as healthy participants. Interestingly, insula activations during fear-conditioned responses may represent a potential predictor biomarker of response to CBT for OCD.

**Disclosure:**

No significant relationships.

